# Can cultural tourism experience enhance cultural confidence? The evidence from Qingyuan Mountain

**DOI:** 10.3389/fpsyg.2022.1063569

**Published:** 2022-12-19

**Authors:** Jian Lin, Yanxin Kang, Liping Hong, Yijun Huang

**Affiliations:** ^1^Tan Siu Lin Business School, Quanzhou Normal University, Quanzhou, China; ^2^School of Tourism, Liming Vocational University, Quanzhou, China

**Keywords:** cultural involvement, cultural tourism experience, cultural identity, cultural confidence, cultural loyalty

## Abstract

A questionnaire survey was conducted among 600 visitors to the region using Qingyuan Mountain, a 5A picturesque location in Quanzhou City, Fujian Province, as the research site. A total of 489 valid questionnaires were received. The links between cultural involvement, cultural experience, cultural identity, cultural confidence, and cultural loyalty were experimentally examined using a structural equation modeling technique. The results showed that cultural experience was a mediating factor in the processes of the influence of cultural involvement on cultural identity and the influence of cultural involvement on cultural confidence, but the influence of cultural involvement on cultural identity and the influence of cultural involvement on cultural confidence were not supported. The study accordingly condenses theoretical contributions to academia and management insights for businesses.

## Introduction

Culture is the sum of the material and spiritual productive capacity and the material and spiritual wealth created by human beings during social practice, and it provides the resource base for tourism, in which people are in fact learning about culture. Cultural tourism as a social phenomenon emerged after the Second World War; after all, it can improve cultural understanding and people’s understanding as well as help build the economy (Richards, 2018). Cultural tourism as an academic study emerged in the 1980s. Cultural tourism reached a high point in the 1980s and 1990s as it became widely recognized as a good tourism product. There was a proliferation of research on cultural tourism, and different theories and research methods were applied to the field (Smith and Richards, 2013). According to the World Tourism Organization’s Tourism and Culture Synergy Report (2018), 89% of the World Tourism Organization’s member states have included cultural tourism in their tourism development policies and are committed to further developing it. According to the report’s projections, cultural tourists will account for more than 40% of the overall tourism sector in the future (UNWTO, 2018). Now is precisely the point in time when the report predicts the next 5 years of planning. Therefore, the current study on cultural tourism is very relevant and timely.

The study of cultural tourism was first defined by the World Tourism Organization as a form of culturally motivated tourism and is one of the oldest forms of ‘new’ tourism (McKercher, 2020). These are tourism, cultural heritage, the experience and consumption of the product and the visitor. The element of tourism is undoubtedly the most fundamental attribute of cultural tourism, which not only facilitates the preservation and exchange of culture but also promotes economic development, revitalizing culture and sustaining its benefits (Shi et al., 2021). Cultural heritage is one of the most dominant forms of cultural tourism and the hottest tourism product (Seyfi et al., 2020). Heritage tourism has become an important ground for scholars to conduct research on cultural tourism. Experience and consumption are important elements of cultural tourism research; after all, cultural tourism involves subject areas such as economics and management, and the desire to maximize the benefits of cultural tourism while preserving it is shared by stakeholders such as tourism developers, local governments, community residents, and cultural custodians (Ammirato et al., 2021). With the rise of the experience economy, the deep integration of cultural tourism and the experience economy has been consistently recognized by many scholars (Su et al., 2020a; Zhang et al., 2021). The study of tourists is a necessary part of any tourism product, and only if tourists accept, recognize and even recommend the tourism product will it have economic value and be sustainable (Su et al., 2020b). In this new era, the types of cultural tourism products are further enriched, and the boundaries between culture and tourism become increasingly blurred (Yang et al., 2022).

Cultural confidence is a collective cultural identity, belonging and love based on an individual’s deep understanding, acceptance and practice of their own culture. It is a powerful spiritual motivator that leads to the formation of certain value dispositions and can lead to positive behavior (Wan and Rucker, 2013; Ortiz-Ordoñez et al., 2015). And to form one’s cultural confidence, one must first have access to channels and opportunities to learn about excellent traditional culture (Zhao, 2022). Traveling is a good learning opportunity, which not only enables the traveler to broaden his or her horizons and gain insight, but also generates a self-confidence from the inside out through this process (Chen, 2022). And this self-confidence has positive implications for personal growth, external communication and patriotism (Lin et al., 2022). Therefore, exploring the mechanisms by which cultural confidence is generated in tourists and its impact on tourism behavior can provide useful references for other scholars exploring similar topics, as well as providing more business management ideas for tourism business managers and valuable references for the education sector in patriotic education.

This study adopts a positivist paradigm to investigate the psychological feelings of tourists after experiencing cultural tourism, with the aim of understanding the experience of cultural tourism, cultural identity, cultural confidence and cultural loyalty and clarifying the interplay among them. This study is a good complement to the current hot research on cultural confidence, broadening the theoretical outreach of cultural tourism research, further enriching the content of cultural tourism and providing a theoretical basis for other scholars in similar research. In addition, the results of this study have certain management practice implications for tourism management departments and are of reference value for tourism developers to carry out targeted tourism marketing for tourists.

## Literature review and hypotheses

### Cultural involvement

Involvement is the evaluation of the importance and relevance of objects by individuals according to their intrinsic needs, values and interests (Zaichkowsky, 1985). According to this definition, cultural involvement can be thought of as tourists evaluating cultural tourism activities and consumption based on their own needs, values and interests (Campos et al., 2017). Carlson and Güler (2018) argue that cultural involvement includes both the culture of origin and destination culture dimensions, while Gao et al. (2020) argue that cultural involvement includes three dimensions, namely, attraction, self-expression, and centrality, and Whang et al. (2016) argue that cultural involvement includes situational involvement and persistent involvement. The study by Jian et al. (2019) used a single dimension of persistent involvement. This study also adopts the concept of a single dimension of persistent involvement.

In a study conducted by Li et al. (2021) on residents’ attitudes toward tourism, cultural involvement was found to have a positive and significant effect on the cultural experience. Whang et al. (2016) argued that cultural transmission is a prerequisite for cultural involvement and that tourists’ experiences cannot be separated from cultural transmission. Lee and Chang (2017) found that cultural involvement was an antecedent variable for the cultural tourism experience in a survey of 901 tourists who visited Taiwan for diet tourism and found that cultural involvement was an antecedent variable of the cultural tourism experience. Lee and Chang’s (2017) study reconfirmed this result.

Jian et al.’s (2019) study found that cultural involvement has a positive impact on cultural identity, such as manifesting a love for a culture or becoming a fan of a culture. Similar results were confirmed in Koenig-Lewis et al.’s (2021) study, in which 1,335 tourists were interviewed, confirming not only the influence of cultural involvement on cultural identity but also the relationship between cultural involvement and cultural experience. Carlson and Güler (2018) confirmed the same results in their study of immigrants as survey respondents.

Cultural confidence is a collective cultural identity, a sense of belonging and love based on an individual’s deep understanding, acceptance and practice of his or her own culture (Pan et al., 2021). Guan et al. (2020) found that cultural involvement directly influenced students’ cultural confidence in a study on the extent of their red cultural identity in Hebei Province. Li et al. (2021) suggested that local governments and communities organize more cultural-themed activities to provide more opportunities for cultural exchange and enhance residents’ cultural involvement, thereby gaining more cultural confidence and cultural identity. At local cultural festivals, it was found that the cultural involvement of visitors was reinforced and helped them enhance their ethnic pride and cultural confidence.

Based on the above findings, this study makes the following hypotheses:

*H1*: Cultural involvement positively influences cultural experience.

*H2*: Cultural involvement positively influences cultural identity.

*H3*: Cultural involvement positively influences cultural confidence.

### Cultural experience

Cultural experiences are generally defined as trips to cultural tourism destinations for the purpose of acquiring knowledge and authentic experiences (Crompton and McKay, 1997), a view that is related to that of Kim et al. (2009), who found that learning knowledge and authentic experiences can be combined into one dimension through their rooted theory research. Kim and Eves (2012) reconfirmed this finding. The single dimension scale of Kim et al. (2009) was also used in this study.

Research on cultural experiences is well documented, with Koenig-Lewis et al. (2021) finding that travelers gained knowledge and enhanced their cultural identity through experiencing local cultural festivals. Similar results hold true for transient expatriates studying abroad, as El-Ouali and Mouhadjer (2019) and Li and Liu (2020) found that international students’ cultural experience of their host country helped them gain a sense of cultural identity and integrate into the local cultural life as soon as possible. In short, cultural identity is constructed on the basis of cultural experience (Gao, 2021).

In terms of exploring the relationship between cultural experience and cultural confidence, Chen’s (2020) study found that university students’ creative design experiences enriched their knowledge of that culture and built up stronger cultural confidence. Mei (2022) found similar results through a study of university students’ experiences of their local culture, which had a significant effect on their cultural confidence.

Chen and Rahman (2018) interviewed tourists involved in cultural tourism and found correlations between engagement, cultural exposure, memorable travel experiences and loyalty to cultural tourism destinations, with memorable travel experiences being positively correlated with cultural tourism destinations. Ogunnaike et al. (2022) studied hotels and found that the cultural ambience of a hotel was crucial in attracting customers, as their cultural experience with the hotel directly influenced their loyalty to the hotel. Suhartanto et al. (2018) studied tourist loyalty using cultural tourism destinations and similarly found that the cultural experience of tourists had a significant direct impact on destination loyalty.

Based on the above findings, this study makes the following hypotheses:

*H4*: Cultural experience positively influences cultural identity.

*H5*: Cultural experience positively influences cultural confidence.

*H6*: Cultural experience positively influences cultural loyalty.

### Cultural identity

Bhugra (2004) argues that cultural identity includes an individual’s adherence to and identification with elements of religion, language, customs, beliefs, rituals, and leisure activities. From the perspective of tourism, tourism contributes to the spread of culture, facilitates communication and brings in funds for the preservation of culture, and in this sense, tourism contributes to the cultural identity of tourists. Luo et al. (2019) conducted a study with tourists visiting the 21st Century Maritime Silk Road Museum and found that visitors who experienced the museum had a high cultural identity, and a strong study by Gao (2021) found that enhancing students’ national cultural identity helped build students’ national cultural self-confidence. Tian et al. (2020) conducted a study on tourists who participated in intangible cultural heritage experiences and found that tourists’ cultural identity would influence cultural destination loyalty through authenticity. Le and Le (2020) conducted a study of tourists visiting Thanh Hoa Province in Vietnam and obtained similar results. In addition to this finding for tourists, Lee et al. (2021) found similar results for Aboriginal people, whose identification with indigenous culture directly influenced their loyalty to that culture.

Based on these findings, this study makes the following hypotheses:

*H7*: Cultural identity positively influences cultural confidence.

*H8*: Cultural identity positively influences cultural loyalty.

### Cultural confidence

Pan et al. (2021), based on previous definitions of self-confidence and through focus interviews with cultural and tourism experts, define cultural confidence as a collective cultural identity, belonging and love that is based on an individual’s deep understanding, acceptance and practice of his or her own culture. As the concept of cultural confidence has emerged only in recent years, research on cultural confidence is not yet very rich and currently focuses more on the integration of culture and identity. Zang and Liu (2021) found through their study of mobile learning among university students that cultural confidence among university students helped to enhance their sense of cultural loyalty. Li’s (2022) study found that Jia et al. (2022) argued that traditional culture should be included in the science and technology courses of university students because it has a direct impact on enhancing their cultural confidence and has a significant effect on their loyalty to their motherland and people, i.e., the enhancement effect. In light of this, this study argues the following:

*H9*: Cultural confidence positively influences cultural loyalty.

### Cultural loyalty

Cultural loyalty is an authority that derives from the voluntary allegiance of its members (Parekh, 2001). Cultural loyalty is a very strong sense of people’s loyalty to their values, ideal beliefs and religious beliefs. It should include at least three voices, namely, the voice of ancestors, the voice of relationships and the voice of ethics (Piquemal, 2005). Loyalty is an important concept that has received widespread scholarly attention, including national loyalty, ethnic loyalty and brand loyalty, among others. Cultural loyalty has received more widespread scholarly attention in recent years because it is more of an intangible soft power and a powerful emotional force, and some scholars have even proposed a cultural loyalty approach (Karkabi, 2021). It is clear that an in-depth study of cultural loyalty is necessary and important.

**Figure 1:**
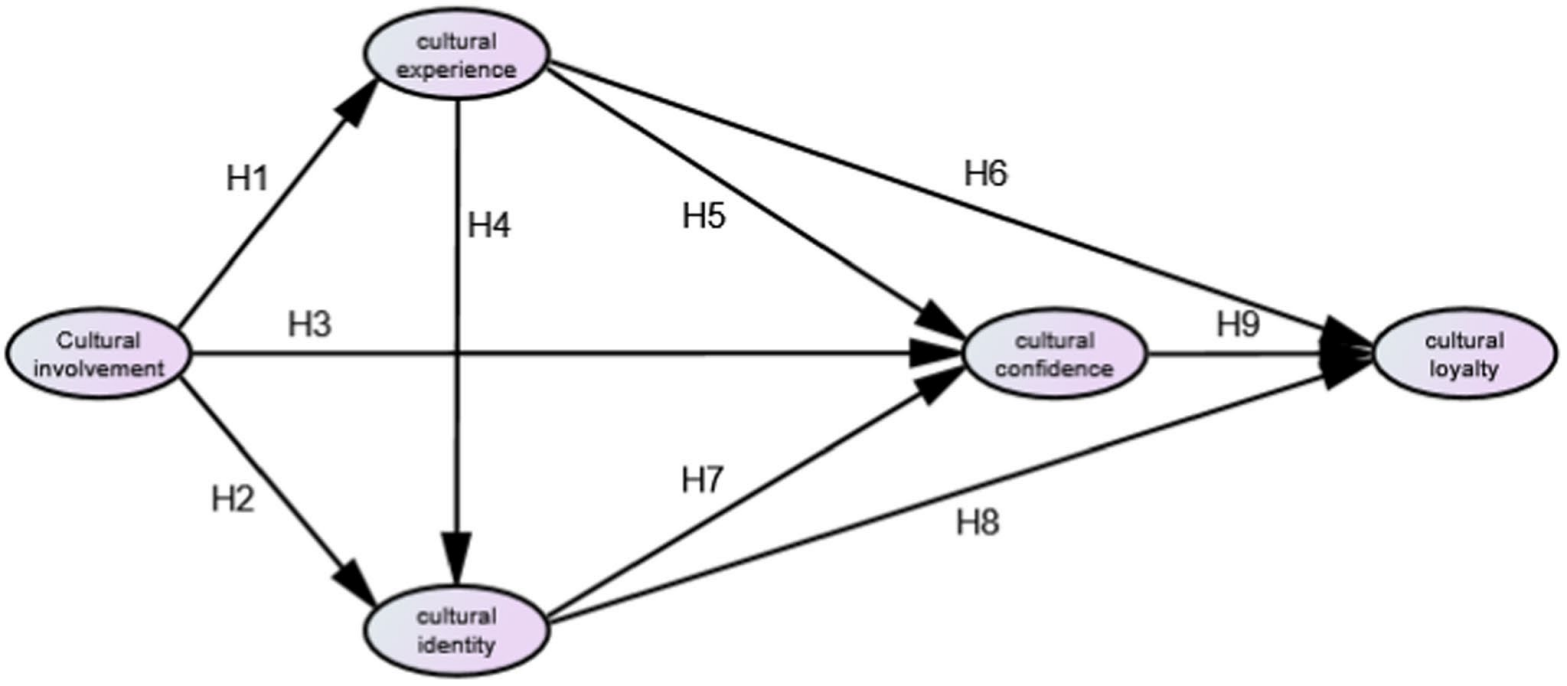
The structure model.

Based on the above research findings and the comprehensive reasoning of this study, the following model is constructed in this study (see Figure 1):

## Materials and methods

### Study area

Located in southeastern Fujian Province, on the northeastern bank of the lower reaches of the Jinjiang River, Qingyuan Mountain is one of the eighteen scenic spots in Quanzhou and a national key scenic spot, consisting of three large areas: Qingyuan Mountain, Nine-Day Mountain and the Holy Tomb of Lingshan, with a total area of sixty-two square kilometers. It is a national 5A level tourist attraction in China. Qingyuan Mountain is a must-see attraction when visiting Quanzhou. The most famous statue of Laojun from the Song Dynasty is the Laojun Rock in the scenic area, which is the largest and most artistically valuable Taoist stone sculpture in China. The stone carving of praying for the wind at Jiuriyama is a precious source for studying the history of overseas transportation and the art of calligraphy in ancient China. The Holy Sepulchre is the place where the three sages and four sages, disciples of Muhammad, came to Quanzhou to preach and were buried, called the Islamic Holy Sepulchre. Qingyuan Mountain is not only beautiful but also an important cultural tourism destination. The number of visitors to Qingyuan Mountain is very high every year, peaking at 30,000 visitors per day. As the only 5A scenic spot in Quanzhou, it has become, to a certain extent, one of Quanzhou’s calling cards for external publicity and an important part of the composition of Quanzhou’s tourism image. Qingyuan Mountain has also become a commonly chosen case study site for local scholars engaged in cultural research, as well as attracting many scholars from around the world who are studying Quanzhou culture, Hokkien culture and the historical traces of Islamic culture. The study has therefore chosen this study site as a representative and viable one.

### Scale design

This study used a self-statement scale for data collection, consisting of six parts, using a 7-point Likert scale design. The first section is cultural involvement, from a study by Jian et al. (2019), the second section is cultural experience, from a study by Kim and Eves (2012), the third section is cultural identity, from a study by He and Wang (2015), the fourth section is cultural confidence, from a study by Pan et al. (2021), the fifth section is cultural loyalty, from a study by Yang and Peterson (2004), and the sixth part is demographic information.

### Data collection

This study was conducted from September 10 to September 13, 2022, at the leisure hall at the exit of the main gate of the Qingyuan Mountain Scenic Area to interview visitors who had finished their visit. Two hundred copies were distributed each day, 100 in the morning and 100 in the afternoon. The research started from the first visitor returning in the morning until 100 copies were collected, and in the afternoon, due to the hot weather, there were many guests resting in the hall, so in order not to disturb the guests’ rest, the survey started from 3 o’clock until 100 copies were collected. A total of 600 questionnaires were distributed and 517 were returned, of which 489 were valid. The visitors were 259 men (52.97%) and 230 women (47.03%). In terms of age composition, 64 people (13.09%) were aged 18-25, 121 people (24.74%) were aged 26-35, 153 people (31.29%) were aged 36-45, 95 people (19.43%) were aged 46-55, 45 people (9.20%) were aged 56-65 and 11 (2.25%) were aged 66 or above. Regarding the composition of education levels, 132 (26.99%) were high school and below, 128 (26.18%) were college, 161 (32.92%) were bachelor’s degree and 68 (13.91%) were master’s degree and above.

## Results

SPSS 24.0 was used to test the quality of the dataset, and the analysis revealed that there were no missing values. The data had a skewness of 3 < and a kurtosis of <7, which basically met the requirements of a normal distribution. The reliability of the variables ranged from 0.845 to 0.918, all reaching the recommended value of >0.7 (see Table 1), and the corrected item total correlation (CITC) between the variables all reached the recommended value of >0.5. The data are of good quality and ready for the next step of analysis.

**Table 1:** The results of confirmatory factor analysis (*n* = 489).

Construct/Items		Std.	T-value	*α*	CR	AVE
Cultural involvement				0.845	0.846	0.578
Cin1	I love and enjoy this culture	0.785				
Cin3	I care a lot about this culture	0.704	15.106			
Cin4	I am particularly interested in this culture	0.743	15.971			
Cin5	I feel like I have a special affinity for this culture	0.806	17.16			
Cultural experience				0.872	0.872	0.694
Ce4	Through travel experience, I can understand the cultural connotation of these scenic spots	0.826				
Ce5	I have the opportunity to increase my understanding of different cultures through travel experience	0.798	19.251			
Ce6	Traveling in a place where the culture is fully preserved is an authentic experience	0.874	20.835			
Cultural identity				0.865	0.866	0.565
Cid1	I am proud of our culture.	0.703				
Cid2	I have admiration for important figures in our nation’s history.	0.671	13.567			
Cid3	Chinese highlight events of historical and cultural importance	0.774	15.487			
Cid4	Our country has a profound historical and cultural heritage.	0.841	16.566			
Cid5	As a Chinese, I think we have certain cultural attributes that other countries do not have.	0.759	15.212			
Cultural confidence				0.860	0.861	0.607
Cc4	The site is well preserved, inheriting the local cultural and historical landscape.	0.764				
Cc3	All the folklore activities and festivals in this place can reflect the essence of local culture.	0.782	16.826			
Cc2	The local culture can help me think and solve the problems I encounter.	0.794	17.072			
Cc1	The culture of the place can guide me to make an accurate self-evaluation.	0.777	16.718			
Cultural loyalty				0.918	0.919	0.694
Cl1	I will say positive things about the culture to others.	0.832				
Cl2	I would recommend this local culture to those who come to me for advice.	0.824	21.713			
Cl3	I would encourage relatives and friends to experience the culture.	0.868	23.498			
Cl4	I will publish positive information about this culture on some Internet platforms (such as WeChat, Weibo, etc.).	0.847	22.629			
Cl5	I intend to continue experiencing the culture.	0.792	20.461			

std., standard estimate; CR, composite reliability.

### Common method bias

To exclude the effect of common method bias, the one-way validation method of Harman (1976) was used in this study. That is, a factor analysis was performed on the dataset to check the cumulative explained variance of the first factor without rotation, and if it was below 50%, the common method bias of the dataset was not serious enough for further analysis. By testing, the cumulative explained variance of the first factor in this study was 25.322%, well below the recommended value of 50%.

### Measurement model

Using Amos 24.0 for validated analysis of the dataset, the measurement model met the recommended values recommended by academics for the model fit indicators, except for AGFI, which was slightly below the recommendation of 0.9 (see Table 2; CFA). The factor loadings for each variable ranged from 0.671 to 0.874, with all questions reaching above 0.7 except for the Cid2 question item, which was slightly below 0.7; the reliability of each variable ranged from 0.846 to 0.919, meeting the scholarly recommendation of greater than 0.7; and the AVE ranged from 0.565 to 0.694, all meeting the recommendation of greater than 0.5. This study used Fornell and Larcker (1981) and Bagozzi and Yi (2012) recommended discriminant validity method to validate the dataset for discriminant validity, and the results showed that the discriminant validity met the criteria recommended by scholars (see Table 3).

**Table 2:** The results of model fit measures.

Index	*χ* ^2^	Df	*χ*^2^/df	RMSEA	GFI	AGFI	CFI	NFI	RFI	IFI	TLI
CFA	445.617	179.000	2.489	0.055	0.918	0.895	0.953	0.925	0.912	0.954	0.945
Structural model	466.839	180.000	2.594	0.057	0.915	0.891	0.950	0.921	0.908	0.950	0.942
Fitted value	–	–	<3.0	<0.05	>0.9	>0.9	>0.9	>0.9	>0.9	>0.9	>0.9

**Table 3:** The discriminant validity.

	CR	AVE	CIn	CE	Cid	CC	CL
Cultural involvement (Cin)	0.846	0.578	**0.761**				
Cultural experience (Ce)	0.872	0.694	0.256	**0.833**			
Cultural identity (Cid)	0.866	0.565	0.067	0.246	**0.752**		
Cultural confidence (Cc)	0.861	0.607	0.105	0.351	0.441	**0.779**	
Cultural loyalty (Cl)	0.919	0.694	0.333	0.558	0.346	0.435	**0.833**

The square root of the AVE is presented in bold.

### Structural equation modeling

**Figure 2:**
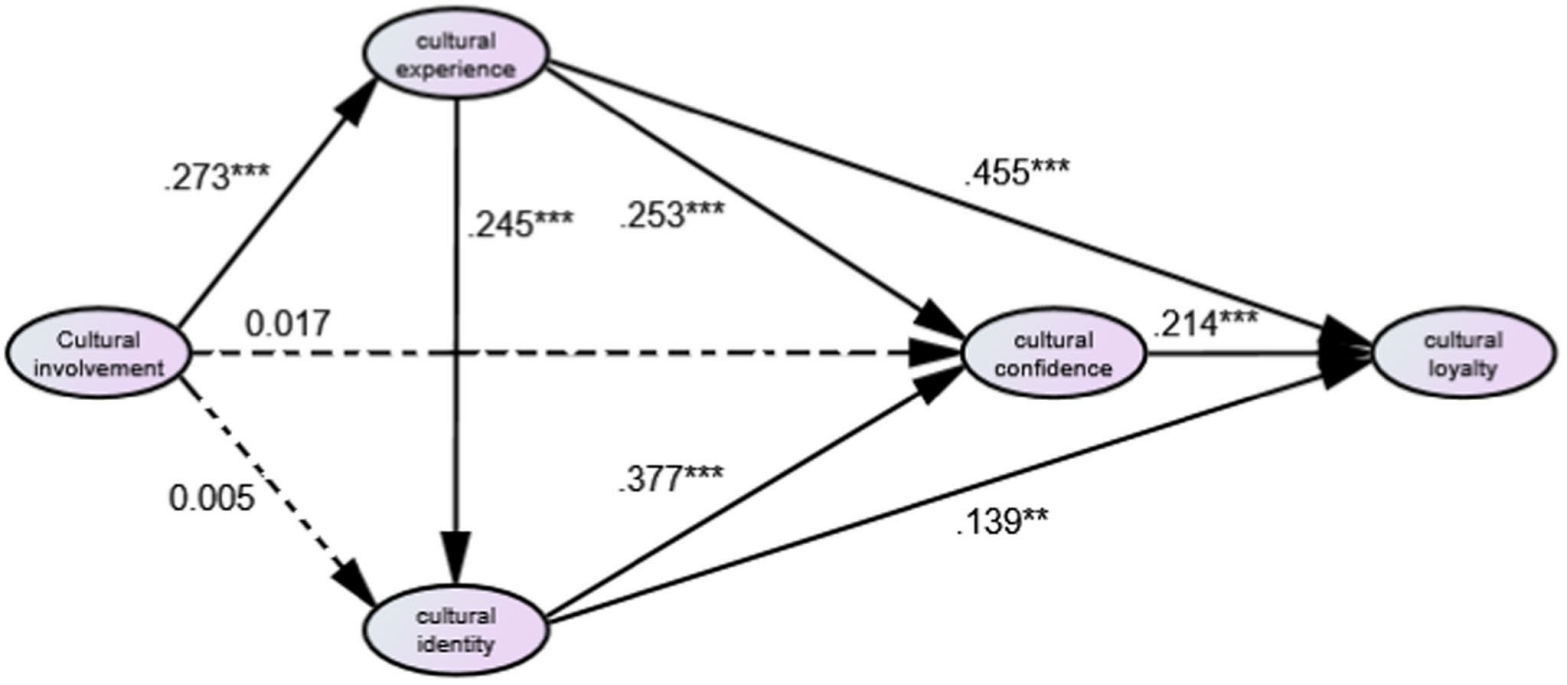
Structure model and path coefficient. *p < 0.05,**p < 0.01, ***p < 0.001.

Structural equation modeling was conducted using the maximum likelihood method of Amos 24.0. The model fit was showed in the table 2-structural. And the results of the path analysis were significant for all the paths except H2: cultural involvement has a positive impact on cultural identity and H3: cultural involvement has a positive impact on cultural confidence, which were not significant (see Figure 2; Table 4).

**Table 4:** Structure parameter estimates.

Hypothesis		Estimate	Std	S.E.	*T*-Value	*p*	Result
H1	Cultural involvement → Cultural experience	0.292	0.273	0.056	5.191	***	Yes
H2	Cultural involvement → Cultural identity	0.005	0.005	0.057	0.084	0.933	No
H3	Cultural involvement → Cultural confidence	0.018	0.017	0.051	0.35	0.726	No
H4	Cultural experience → Cultural identity	0.243	0.245	0.054	4.477	***	Yes
H5	Cultural experience → Cultural confidence	0.241	0.253	0.05	4.825	***	Yes
H6	Cultural experience → Cultural loyalty	0.488	0.455	0.053	9.299	***	Yes
H7	Cultural identity → Cultural confidence	0.363	0.377	0.052	7.033	***	Yes
H8	Cultural identity → Cultural loyalty	0.151	0.139	0.052	2.882	0.004	Yes
H9	Cultural confidence → Cultural loyalty	0.241	0.214	0.058	4.184	***	Yes

std., standard estimate. *p < 0.05, **p < 0.01, ***p < 0.001.

### Mediating effects

As the two paths of cultural involvement on cultural identity and cultural involvement on cultural confidence are not significant, and cultural experience is in the intermediary position between the two paths of cultural involvement and cultural identity and cultural involvement and cultural confidence, it is necessary to explore the mediating effect of cultural experience to further explore the relationships between cultural involvement and cultural identity and cultural involvement and cultural confidence. This paper uses the bootstrap (bootstrap = 2000) method to further verify the mediating effect of cultural experiences. The results are shown in Table 5 below, where cultural experiences play a mediating role in the pathways of the influence of cultural involvement on cultural identity and cultural involvement on cultural confidence.

**Table 5:** Mediation effect.

Path		Point estimate	Product of coefficients		Percentile 95%CI		Bias-corrected percentile 95%CI		Mediation
			se	*Z*-value	Lower	Upper	Lower	Upper	
Cin → Cid	Total effects	0.076	0.060	1.267	−0.054	0.191	−0.055	0.191	
	Direct effects	0.005	0.062	0.081	−0.119	0.124	−0.121	0.123	
Cin → CE → Cid	Indirect effects	0.071	0.026	2.731	0.024	0.126	0.026	0.135	Yes
Cin → CC	Total effects	0.116	0.068	1.706	−0.017	0.25	−0.009	0.259	
	Direct effects	0.018	0.052	0.346	−0.085	0.115	−0.082	0.117	
Cin → CE → CC	Indirect effects	0.098	0.042	2.333	0.021	0.188	0.026	0.197	Yes

Cin, Cultural involve; CE, Cultural experience; Cid, Cultural identity.

## Discussion and conclusion

### Discussion

This study takes the famous religious mountain located in Quanzhou City, Fujian Province, as an example and explores the relationships between cultural involvement, cultural experience, cultural identity, cultural confidence and cultural loyalty. The results showed that most of the hypotheses were tested, except for the influence of cultural involvement on cultural identity and the influence of cultural involvement on cultural confidence, which were not tested. In addition, the mediating role of cultural experience in the influence of cultural involvement on cultural identity and the influence of cultural involvement on cultural confidence was also tested.

From the results, the relationship between cultural involvement and the influence of cultural experience was verified, which is consistent with previous research (Gao, 2021; Koenig-Lewis et al., 2021). After all, the higher the degree of cultural involvement, the more likely it is that the motivation to experience is generated, and once a person has generated motivation, it is easy to put it into action, that is, to produce the act of experiencing. When travellers have a cultural experience, they are bound to have certain feelings about the culture, which may be good or bad. If it is a good feeling, they are bound to appreciate, identify with or even love the culture, while if it is a bad feeling, they may reject the culture and fail to integrate into it (Wei et al., 2020). This is common among new immigrants and explains why many immigrants are unable to adapt to the culture of the place they have moved to. In addition to giving identity, experience may also give rise to a sense of confidence and even pride, because culture is powerful and intangible, and culture is also a source of spiritual strength for people, as evidenced in the research at Karadağ et al. (2020). Of course, when a culture has become a person’s spiritual strength, he is bound to cherish that strength and be loyal to that culture without reluctance, which explains the relationship between the influence of cultural involvement and cultural loyalty in this study.

Identity is the identification of people with something, a role or a culture. It is not just an acceptance but a high degree of emotional approval, so that identity is a positive psychological state that gives people positive energy and creates psychological confidence. When a person is highly identified with a culture or even becomes an integral part of his or her own culture, he or she is bound to be loyal to that culture because it is already an important part of his or her culture, psyche and spirit. Therefore, the influence of cultural identity on cultural confidence (Luo et al., 2019; Gao, 2021) and the influence of cultural identity on cultural loyalty (Tian et al., 2020; Lee et al., 2021) in this study are reasonable and in line with previous research.

Self-confidence is an absolutely positive psychological state in human beings; it is a spiritual motivation and source of strength that sustains one’s persistence, effort and perseverance, and this also applies in the field of culture. Therefore, the hypothesis that cultural confidence has a positive impact on cultural loyalty is valid and in line with the results of previous studies (Zang and Liu, 2021; Jia et al., 2022; Li, 2022).

However, the hypothesis that cultural exposure has a positive impact on cultural identity has not been tested, contrary to the findings of previous studies. Cultural involvement is a superficial perception that may come from oral accounts, indirect knowledge from films, television, books, etc., or fragmented knowledge. This kind of knowledge makes it difficult to form a three-dimensional perception in the visitor’s mind, much less to make him or her feel good about it or accept, or even highly value, the culture (Smolicz, 1981). This is therefore a good explanation for why cultural involvement does not hold true for the positive impact of cultural identity. Similarly, when visitors’ perceptions are only at this superficial level, it is not possible for them to develop a sense of confidence. Therefore, it is possible that the effect of cultural involvement on cultural confidence does not hold true. A study by McKercher and Du Cros (2002) found that approximately half of tourists are not influenced by cultural involvement when choosing a tourist destination.

It is of interest to note that cultural experience has an important place in this study, as it plays an extremely important role in the overall mechanism of cultural influence (Armbrecht, 2014; Lembo and Martin, 2022). The influence of cultural involvement on cultural experience is present, as is the influence of cultural experience on cultural identity, cultural confidence and cultural loyalty. In addition to this, cultural experiences play a mediating role in the influence of cultural involvement on cultural identity, as well as in the influence of cultural involvement on cultural confidence. It is clear that it is difficult to develop cultural tourism without well-designed experiences to create identity and confidence in visitors, which explains exactly why the experience economy is so hot and the importance of experience quality in the tourism product.

### Theoretical implications

This study enriches the theory of cultural tourism research. Cultural tourism has traditionally been a key area of research in the tourism sector, receiving much attention not only from tourism scholars but also from cultural scholars. However, it is rare to integrate cultural involvement, cultural experience, cultural identity, cultural confidence and cultural loyalty into one study, and it is also rare to conduct in-depth research and discussion on their interrelationships and mechanisms of influence. Therefore, the attempt of this study provides more references for subsequent research on cultural tourism.

The two hypotheses found to be unsupported in this study add to the previous research. Unlike in previous studies, the impact of cultural involvement on cultural identity and the impact of cultural involvement on cultural confidence are not supported in this study. This suggests that there are still some gaping points in the previous study and further clarifies the relationships among the three, which is also an important finding in this respect.

The two complete mediators found in this study further confirm the importance of cultural experience. Cultural experience has been extensively and thoroughly researched in previous studies, but its importance, especially in the field of cultural tourism, should be given more attention by scholars, as after all, tourists’ cultural identity and cultural confidence are based on good cultural experience. This result also provides a theoretical reference for other scholars to carry out similar studies in the future.

### Implications for management practice

Cultural tourism development should pay attention to tourism promotion. The basis for a tourist’s motivation to travel is that he has some knowledge of this tourist destination. When information about a tourist destination is not disseminated to the minds of potential tourists, or when tourists are simply unaware of the existence of such a tourist destination or tourist product, they cannot be motivated to travel. Therefore, in the process of cultural tourism development, it is important to strengthen publicity efforts, broaden publicity channels, enrich publicity methods and focus on the effects of publicity. After all, in the current era of diverse information dissemination channels, the amount of information is exploding, and without timely and effective publicity, it is easily buried by new information.

Cultural tourism development should pay attention to experience design and improve the quality of the experience. The experience economy has been here for a long time, and the importance of experience marketing for products has become an irrefutable marketing approach to business management. However, previous experience marketing focused on tangible products, and after all, it can give people a real sense of presence; however, cultural products, including cultural tourism, are intangible products, and passive preaching has been unable to move the emotions of tourists or make them more likely to resonate. Therefore, intangible cultural tourism should pay more attention to the experience process and experience quality.

Cultural tourism development should strive to gain the recognition of tourists and work hard to create cultural confidence in them to form cultural loyalty. Whereas products bring limited benefits to an enterprise, brands bring unlimited, long-term benefits. Cultural tourism products can also be a good brand, but it is essential that visitors identify with the product and brand and develop national cultural self-confidence in it. Only in this way is it possible for visitors to develop loyalty to the cultural tourism brand, which is very useful and necessary to develop customer stickiness.

## Research limitations and future study

Due to the openness of the study site and the human and material resource constraints of this study, this study adopts a convenience sampling method; the form of the data is cross-sectional, and the representativeness may be somewhat different from that of the random sampling method. Second, the case study site for this study is Qingyuan Mountain in Quanzhou City, Fujian Province. There are many other famous mountains and rivers with the same profound cultural heritage, and geographical differences may lead to cultural differences; therefore, the results of this study should be taken with caution when generalizing to other study sites.

Cultural tourism is a big topic that needs more scholars to be involved in it and more variables to be tapped to study the field of cultural tourism thoroughly and to provide a truly useful reference for the theoretical and industrial communities. Therefore, in future research, consideration could be given to adding contingent variables such as perceived value, cultural consistency, authenticity, etc., as well as moderating variables and multicluster analysis for different groups of people, to clarify the influence mechanisms of cultural tourism.

## Data availability statement

The original contributions presented in the study are included in the article/supplementary material, further inquiries can be directed to the corresponding author.

## Author contributions

JL and YH conceived the study. JL, YK, LH, and YH wrote the manuscript. All authors designed the study, collected and analyzed the data, read and approved the manuscript, and agreed to be accountable for all aspects of the work.

## Funding

This paper was supported by Innovation Strategy Research Plan project of Fujian Provincial Science and Technology Department, “Research on Innovation ecosystem Construction of Digital Creative Industry” (2021R0120), and Education and Scientific Research Project of Young and Middle-aged Teachers in Fujian Province (Social Sciences) General project: Research on the development path of old-age tourism industry in Quanzhou City under the background of aging (JAS21638).

## Conflict of interest

The authors declare that the research was conducted in the absence of any commercial or financial relationships that could be construed as a potential conflict of interest.

## Publisher’s note

All claims expressed in this article are solely those of the authors and do not necessarily represent those of their affiliated organizations, or those of the publisher, the editors and the reviewers. Any product that may be evaluated in this article, or claim that may be made by its manufacturer, is not guaranteed or endorsed by the publisher.

## References

[ref1] Ammirato S. Felicetti A. M. Linzalone R. Carlucci D. (2021). Digital business models in cultural tourism. Int. J. Entrep. Behav. Res. 28, 1940–1961. doi: 10.1108/IJEBR-01-2021-0070

[ref2] Armbrecht J. (2014). Use value of cultural experiences: a comparison of contingent valuation and travel cost. Tour. Manag. 42, 141–148. doi: 10.1016/j.tourman.2013.11.010

[ref3] Bagozzi R. P. Yi Y. (2012). Specification, evaluation, and interpretation of structural equation models. J. Acad. Mark. Sci. 40, 8–34. doi: 10.1007/s11747-011-0278-x, PMID: 36293859

[ref4] Bhugra D. (2004). Migration, distress and cultural identity. Br. Med. Bull. 69, 129–141. doi: 10.1093/bmb/ldh007, PMID: 15226202

[ref5] Campos A. C. Mendes J. do Valle P. O. Scott N. (2017). Co-creating animal-based tourist experiences: attention, involvement and memorability. Tour. Manag. 63, 100–114. doi: 10.1016/j.tourman.2017.06.001

[ref6] Carlson E. Güler A. (2018). Cultural involvement and preference in immigrant acculturation. J. Int. Migr. Integr. 19, 625–647. doi: 10.1007/s12134-018-0554-4, PMID: 34637476

[ref7] Chen J. (2020). Research on the cultivation of college students’ design originality from the perspective of cultural confidence. J. Contemp. Educat. Res. 4, 47–49. doi: 10.26689/jcer.v4i10.1564

[ref8] Chen D. (2022). Study on the ways to integrate cultural confidence in the new era into the ideological and political class of college students. Int. J. Soc. Sci. Educat. Res. 5, 238–243. doi: 10.47205/jdss.2021(2-iv)74

[ref9] Chen H. Rahman I. (2018). Cultural tourism: an analysis of engagement, cultural contact, memorable tourism experience and destination loyalty. Tour. Manag. Perspect. 26, 153–163. doi: 10.1016/j.tmp.2017.10.006

[ref10] Crompton J. L. McKay S. L. (1997). Motives of visitors attending festival events. Ann. Tour. Res. 24, 425–439. doi: 10.1016/S0160-7383(97)80010-2, PMID: 34263390

[ref11] El-Ouali F. Z. Mouhadjer N. (2019). Cultural identity reconstruction in the study abroad context: the case of Algerian Sojourners. Glob. J. Foreign Lang. Teach. 9, 214–225. doi: 10.18844/gjflt.v9i4.4366

[ref12] Fornell C. Larcker D. F. (1981). Evaluating structural equation models with unobservable variables and measurement error. J. Mark. Res. 18, 39–50. doi: 10.1177/002224378101800104, PMID: 33691717

[ref13] Gao F. (2021). Negotiation of native linguistic ideology and cultural identities in English learning: a cultural schema perspective. J. Multiling. Multicult. Dev. 42, 551–564. doi: 10.1080/01434632.2020.1857389

[ref14] Gao J. Lin S. S. Zhang C. (2020). Authenticity, involvement, and nostalgia: understanding visitor satisfaction with an adaptive reuse heritage site in urban China. J. Destin. Mark. Manag. 15:100404. doi: 10.1016/j.jdmm.2019.100404

[ref15] Guan C. Xiao L. Ma W. (2020). The status quo and problem analysis of Hebei red cultural value identification by English majors in Hebei Province. Open J. Modern Linguist. 10, 125–131. doi: 10.4236/ojml.2020.102008

[ref16] Harman H. H. (1976). Modern Factor Analysis. Chicago Horace: University of Chicago Press.

[ref17] He J. Wang C. L. (2015). Cultural identity and consumer ethnocentrism impacts on preference and purchase of domestic versus import brands: an empirical study in China. J. Bus. Res. 68, 1225–1233. doi: 10.1016/j.jbusres.2014.11.017

[ref18] Jia X. Miao W. Yu D. Liu H. He C. (2022). Chinese culture integrated into the science and engineering courses. Sci. Soc. Res. 4, 1–6. doi: 10.26689/ssr.v4i6.3977, PMID: 34366272

[ref19] Jian Y. Zhou Z. Zhou N. (2019). Brand cultural symbolism, brand authenticity, and consumer well-being: the moderating role of cultural involvement. J. Product Brand Manag. 28, 529–539. doi: 10.1108/JPBM-08-2018-1981

[ref01] Karadağ M. Altınay Aksal F. Altınay Gazi Z. Dağli G. (2020). Effect size of spiritual leadership: in the process of school culture and academic success. Sage Open 10. doi: 10.1177/2158244020914638

[ref21] Karkabi N. (2021). The impossible quest of Nasreen Qadri to claim colonial privilege in Israel. Ethn. Racial Stud. 44, 966–986. doi: 10.1080/01419870.2021.1877314

[ref22] Kim Y. G. Eves A. (2012). Construction and validation of a scale to measure tourist motivation to consume local food. Tour. Manag. 33, 1458–1467. doi: 10.1016/j.tourman.2012.01.015

[ref23] Kim Y. G. Eves A. Scarles C. (2009). Building a model of local food consumption on trips and holidays: a grounded theory approach. Int. J. Hosp. Manag. 28, 423–431. doi: 10.1016/j.ijhm.2008.11.005

[ref24] Koenig-Lewis N. Palmer A. Asaad Y. (2021). Linking engagement at cultural festivals to legacy impacts. J. Sustain. Tour. 29, 1810–1831. doi: 10.1080/09669582.2020.1855434

[ref26] Le H. B. H. Le T. B. (2020). Impact of destination image and satisfaction on tourist loyalty: mountain destinations in Thanh Hoa province, Vietnam. J. Asian Finan. Econ. Bus. 7, 185–195. doi: 10.13106/jafeb.2020.vol7.no4.185

[ref27] Lee T. H. Chang P.-S. (2017). Examining the relationships among festivalscape, experiences, and identity: evidence from two Taiwanese aboriginal festivals. Leis. Stud. 36, 453–467. doi: 10.1080/02614367.2016.1190857

[ref28] Lee T. H. Lin Y. H. Wang C.-K. (2021). Can aboriginal images contribute to aboriginal cultural identity? Evidence from the perspective of tourists’ images. Curr. Issue Tour. 25, 1–16. doi: 10.1080/13683500.2021.2005553

[ref29] Lembo A. Martin J. L. (2022). The structure of cultural experience. Poetics 91:101562. doi: 10.1016/j.poetic.2021.101562, PMID: 36424711

[ref30] Li J. (2022). The positive influence of traditional classical literature Reading promotion activities on mental heath. Forest Chem. Rev. 653–664.

[ref31] Li Y.-Q. Liu C.-H. (2020). Impact of cultural contact on satisfaction and attachment: mediating roles of creative experiences and cultural memories. J. Hosp. Market. Manag. 29, 221–245. doi: 10.1080/19368623.2019.1611516

[ref32] Li J. Pan L. Hu Y. (2021). Cultural involvement and attitudes toward tourism: examining serial mediation effects of residents’ spiritual wellbeing and place attachment. J. Destin. Mark. Manag. 20:100601. doi: 10.1016/j.jdmm.2021.100601

[ref33] Lin G. Hao L. Yong W. (2022). A study on the value of cultural self-confidence cultivated by the rise of. Acad. J. Humanit. Soc. Sci. 5, 29–34. doi: 10.25236/ajhss.2022.050506

[ref34] Luo Y. Chu D. Xing D. Pan J. Huang Y. Yu J. (2019). On museum tourism development from the perspective of cultural identity experience—taking the core area of the 21st century maritime silk road as an example. Paper presented at the 2nd International Seminar on Education Research and Social Science (ISERSS 2019).

[ref35] McKercher B. (2020). Cultural tourism market: a perspective paper. Tour. Rev. doi: 10.1108/TR-03-2019-0096

[ref36] McKercher B. Du Cros H. (2002). Cultural Tourism: The Partnership between Tourism and Cultural Heritage Management. New York: Haworth Hospitality Press.

[ref37] Mei T. (2022). New trends of culture response in contemporary China: a case study of cultural localization. Paper presented at the 2021 International Conference on Social Development and Media Communication (SDMC 2021).

[ref38] Ogunnaike O. O. Agada S. A. Ighomereho O. S. Borishade T. T. (2022). Social and cultural experiences with loyalty towards hotel services: the mediating role of customer satisfaction. Sustainability 14:8789. doi: 10.3390/su14148789

[ref39] Ortiz-Ordoñez J. C. Stoller F. Remmele B. (2015). Promoting self-confidence, motivation and sustainable learning skills in basic education. Procedia Soc. Behav. Sci. 171, 982–986. doi: 10.1016/j.sbspro.2015.01.205, PMID: 27409075

[ref40] Pan L. Xu X. a. Lu L. Gursoy D. (2021). How cultural confidence affects local residents’ wellbeing. Serv. Ind. J. 41, 581–605. doi: 10.1080/02642069.2018.1540595, PMID: 27409075

[ref41] Parekh B. (2001). Rethinking multiculturalism: cultural diversity and political theory. Ethnicities 1, 109–115. doi: 10.1177/146879680100100112

[ref42] Piquemal N. (2005). Cultural loyalty: aboriginal students take an ethical stance. Reflective Pract. 6, 523–538. doi: 10.1080/14623940500300707

[ref43] Richards G. (2018). Cultural tourism: a review of recent research and trends. J. Hosp. Tour. Manag. 36, 12–21. doi: 10.1016/j.jhtm.2018.03.005, PMID: 35859832

[ref44] Seyfi S. Hall C. M. Rasoolimanesh S. M. (2020). Exploring memorable cultural tourism experiences. J. Herit. Tour. 15, 341–357. doi: 10.1080/1743873X.2019.1639717

[ref45] Shi Z. Cheng Q. Xu D. (2021). Spatial econometric analysis of cultural tourism development quality in the Yangtze River Delta. Asia Pacif. J. Tour. Res. 26, 597–613. doi: 10.1080/10941665.2021.1886131

[ref46] Smith M. K. Richards G. (2013). The Routledge Handbook of Cultural Tourism. Oxon: Routledge.

[ref47] Smolicz J. (1981). Core values and cultural identity. Ethn. Racial Stud. 4, 75–90.

[ref48] Su X. Li X. Chen W. Zeng T. (2020a). Subjective vitality, authenticity experience, and intangible cultural heritage tourism: an empirical study of the puppet show. J. Travel Tour. Mark. 37, 258–271. doi: 10.1080/10548408.2020.1740141

[ref49] Su X. Li X. Wang Y. Zheng Z. Huang Y. (2020b). Awe of intangible cultural heritage: the perspective of ICH tourists. SAGE Open 10:2158244020941467. doi: 10.1177/2158244020941467

[ref50] Suhartanto D. Clemes M. D. Wibisono N. (2018). How experiences with cultural attractions affect destination image and destination loyalty. Tour. Cult. Communicat. 18, 176–188. doi: 10.3727/109830418X15319363084463

[ref51] Tian D. Wang Q. Law R. Zhang M. (2020). Influence of cultural identity on tourists’ authenticity perception, tourist satisfaction, and traveler loyalty. Sustainability 12:6344. doi: 10.3390/su12166344

[ref52] UNWTO (2018). Report on tourism and cultural synergies. Madrid UNWTO.

[ref53] Wan E. W. Rucker D. D. (2013). Confidence and construal framing: when confidence increases versus decreases information processing. J. Consum. Res. 39, 977–992. doi: 10.1086/666467

[ref54] Wei C. Dai S. Xu H. Wang H. (2020). Cultural worldview and cultural experience in natural tourism sites. J. Hosp. Tour. Manag. 43, 241–249. doi: 10.1016/j.jhtm.2020.04.011

[ref55] Whang H. Yong S. Ko E. (2016). Pop culture, destination images, and visit intentions: theory and research on travel motivations of Chinese and Russian tourists. J. Bus. Res. 69, 631–641. doi: 10.1016/j.jbusres.2015.06.020

[ref56] Yang Z. Peterson R. T. (2004). Customer perceived value, satisfaction, and loyalty: the role of switching costs. Psychol. Mark. 21, 799–822. doi: 10.1002/mar.20030

[ref57] Yang Y. Wang S. Cai Y. Zhou X. (2022). How and why does place identity affect residents’ spontaneous culture conservation in ethnic tourism community? A value co-creation perspective. J. Sustain. Tour. 30, 1344–1363. doi: 10.1080/09669582.2021.1945070

[ref58] Zaichkowsky J. L. (1985). Measuring the involvement construct. J. Consum. Res. 12, 341–352. doi: 10.1086/208520, PMID: 36427831

[ref59] Zang X. Liu X. (2021). Design of vocal music mobile learning platform based on digital information technology. Paper presented at the 2021 4th International Conference on Information Systems and Computer Aided Education.

[ref60] Zhang Q. Liu X. Li Z. Tan Z. (2021). Multi-experiences in the art performance tourism: integrating experience economy model with flow theory. J. Travel Tour. Mark. 38, 491–510. doi: 10.1080/10548408.2021.1952148

[ref61] Zhao X. (2022). Evaluation of the modern value of imperial examination culture in the context of cultural confidence based on deep learning models. Mob. Inf. Syst. 2022:9748146. doi: 10.1155/2022/9748146

